# Geriatric screening, fall characteristics and 3- and 12 months adverse outcomes in older patients visiting the emergency department with a fall

**DOI:** 10.1186/s13049-021-00859-5

**Published:** 2021-03-04

**Authors:** Laura C. Blomaard, Simon P. Mooijaart, Leonie J. van Meer, Julia Leander, Jacinta A. Lucke, Jelle de Gelder, Sander Anten, Jacobijn Gussekloo, Bas de Groot

**Affiliations:** 1 Department of Internal Medicine, section Geriatrics, Leiden University Medical Center, PO Box 9600, 2300 RC Leiden, The Netherlands; 2 Institute of Evidence-Based Medicine in Old Age | IEMO, Leiden, The Netherlands; 3 Department of Emergency Medicine, Spaarne Gasthuis, Haarlem, The Netherlands; 4 Department of Public Health and Primary Care, Leiden University Medical Center, Leiden, The Netherlands; 5 Department of Internal Medicine, section Acute Care, Alrijne Hospital, Leiderdorp, The Netherlands; 6 Department of Emergency Medicine, Leiden University Medical Center, Leiden, The Netherlands

**Keywords:** Aged, Falls, Frailty, Geriatric emergency medicine, Older adults

## Abstract

**Background:**

Falls in older Emergency Department (ED) patients may indicate underlying frailty. Geriatric follow-up might help improve outcomes in addition to managing the direct cause and consequence of the fall. We aimed to study whether fall characteristics and the result of geriatric screening in the ED are independently related to adverse outcomes in older patients with fall-related ED visits.

**Methods:**

This was a secondary analysis of the observational multicenter Acutely Presenting Older Patient (APOP) study, of which a subset of patients aged ≥70 years with fall-related ED visits were prospectively included in EDs of two Dutch hospitals. Fall characteristics (cause and location) were retrospectively collected. The APOP-screener was used as a geriatric screening tool. The outcome was 3- and 12-months functional decline and mortality. We assessed to what extent fall characteristics and the geriatric screening result were independent predictors of the outcome, using multivariable logistic regression analysis.

**Results:**

We included 393 patients (median age 80 (IQR 76–86) years) of whom 23.0% were high risk according to screening. The cause of the fall was extrinsic (49.6%), intrinsic (29.3%), unexplained (6.4%) or missing (14.8%). A high risk geriatric screening result was related to increased risk of adverse outcomes (3-months adjusted odds ratio (AOR) 2.27 (1.29–3.98), 12-months AOR 2.20 (1.25–3.89)). Independent of geriatric screening result, an intrinsic cause of the fall increased the risk of 3-months adverse outcomes (AOR 1.92 (1.13–3.26)) and a fall indoors increased the risk of 3-months (AOR 2.14 (1.22–3.74)) and 12-months adverse outcomes (AOR 1.78 (1.03–3.10)).

**Conclusions:**

A high risk geriatric screening result and fall characteristics were both independently associated with adverse outcomes in older ED patients, suggesting that information on both should be evaluated to guide follow-up geriatric assessment and interventions in clinical care.

**Supplementary Information:**

The online version contains supplementary material available at 10.1186/s13049-021-00859-5.

## Introduction

Falls among older people are common and often result in injuries and Emergency Department (ED) visits, [1, 2] which are associated with adverse outcomes such as ED revisits, functional decline and mortality [3–6]. Even minor injuries can result in functional decline and reduction of quality of life [7]. However, not all older people presenting to the ED with a fall are at high risk of adverse outcomes because they are a very heterogeneous group: some are vital, others have considerable frailty. It is known that frail older patients have high risks of adverse outcomes and therefore several geriatric screening tools have been developed to identify high risk geriatric patients in the ED. [8]

In older people, falls can be a representation of underlying frailty [9]. Falls may also have other causes like extrinsic causes (e.g. traffic accidents), intrinsic causes (e.g. syncope) and unknown causes, which may result in different outcomes [10, 11]. Someone who has tripped over the carpet at home may be at higher risk of poor outcomes than someone who fell outside during cycling [12]. Although fall-related injuries (e.g. hip fracture) have been shown to be associated with adverse outcomes, it is unknown to what extent falls can be attributed to frailty and whether the cause and circumstances of falls are associated with adverse outcomes apart from the result of geriatric screening in the ED. It is possible that some causes or circumstances have a greater impact on short- and long-term outcomes in patients who have a high risk on adverse outcomes according to geriatric screening compared to patients with a low risk. Patients in whom the cause and circumstances of the fall are associated with adverse outcomes may benefit from more comprehensive ED management and geriatric follow-up, whereas for patients in whom the fall is not associated with adverse outcomes standard ED management may be appropriate [13, 14]. It would be more (cost-)effective to use our scarce resources and follow-up for those patients who need it most.

The aim of the present study was therefore to assess whether the result of geriatric screening in the ED and fall characteristics (cause and circumstance of falls) are independently related to 3- and 12-months adverse outcomes in older patients with fall-related ED visits. We hypothesized that the majority of older patients with fall-related ED visits would have a high risk geriatric screening result, and that a high risk screening result would increase the impact of the cause or location of the fall on adverse outcomes.

## Methods

### Study design

This was a pre-planned secondary analysis of the Acutely Presenting Older Patient (APOP) study, a prospective multicenter cohort study which included older patients visiting the EDs of four Dutch hospitals from September 2014 till January 2017 [15, 16]. For the present study, additional data of patients with fall-related ED visits was retrospectively collected from two hospitals: the Leiden University Medical Center, an academic hospital with a level 1 trauma center, and the Alrijne Hospital, a teaching hospital with a level 2 trauma center. The EDs of these hospitals together serve the region of Leiden, including all older patients who need to visit an ED due to a fall. Written informed consent was obtained from all patients. The Medical Ethics Committees of all hospitals approved the study.

### Study participants

In the present study, all consecutive patients aged ≥70 years with a fall-related ED visit were included. Whether the visit was fall-related was obtained by asking patients the question: ‘Is the reason for your ED visit related to a fall?’. Exclusion criteria were triage category ‘red’ on the Manchester Triage System (MTS), [17] patients who were unable to approach due to an unstable medical condition, an impaired mental status (i.e. coma) without an authorized proxy present to provide informed consent, a language barrier or refusal to participate [15].

### Data collection

#### Baseline data

Data was collected on demographics, disease severity and geriatric measurements. Demographics consisted of age, sex and living arrangement. Disease severity included arrival by ambulance, triage urgency according to the MTS, [17] chief complaint, [16] and the treating specialist in the ED. Geriatric measurements consisted of the use of a walking device, the number of self-reported medications (≥5 medications meaning polypharmacy), Katz index of Activities of Daily Living (ADL) score (assessing the functional status 2 weeks before ED presentation) [18] and cognitive impairment assessed with the 6-item Cognitive Impairment Test (6CIT) [19].

#### Geriatric screening

As a geriatric screening tool, the APOP screener was used. The APOP screener is a risk stratification instrument which was developed and validated to identify older ED patients at risk for mortality and/or functional decline within 3 months [16]. The screener comprises seven predictors which are collected in less than 2 min after ED arrival. The result of the APOP screener was retrospectively calculated for patients with fall-related ED visits. In routine ED care, a cut-off point is used to indicate clinicians which older patients are at highest risk of adverse outcomes and therefore need extra care. The APOP screener indicates patients with the highest 20% predicted risk of the composite outcome of mortality and/or functional decline within 3 months. The threshold for a ‘high-risk’ APOP screening result is a predicted risk of 45% or greater [16]. The APOP screener is not a frailty screener per se, which means that high risk patients might not represent the frailty population in general, but there is probably a large overlap.

#### Follow-up data

To obtain data on functional status, patients were contacted by telephone 3 and 12 months after their ED visit. Data on mortality was obtained from municipal records.

#### Fall-related ED visit

Additional fall-related data were retrospectively collected from medical records. If the patient indicated that the ED visit was related to a fall, but the medical record indicated otherwise, the information in the medical file was decisive and the patient was excluded from the analyses.

#### Cause of the fall

The cause of the fall was collected from medical records and categorized into four categories by two independent researchers (LCB and LJvM). In case of disagreement, a third researcher decided upon the final category (BdG). The case selection, variables and fall categories were defined prior to data collection by all researchers (Additional file 1) [20]. The four categories were: extrinsic cause, intrinsic cause, unexplained falls and unknown cause due to missing data. Patients were categorized in the category ‘extrinsic cause’ when the record explicitly stated a mechanical, external cause of the fall, i.e. slipping/tripping or traffic accidents [5]. Patients had an ‘intrinsic cause’ when the record stated a medical reason for the fall, i.e. falls due to cerebrovascular events or syncope [21]. Patients were categorized in ‘unexplained falls’ when they had no recollections of events, when history taking was not possible or when no apparent cause of the fall was stated in the record, yet it was evident that the treating physician searched for a possible explanation [22, 23]. If the medical record provided insufficient data about the cause of the fall, the patient had an unknown cause, which was categorized as ‘missing data’. The result of geriatric screening in the ED was not taken into account during categorization of causes of falls.

#### Circumstances of the fall and fall-related injuries

One researcher (LCB) collected data on circumstances of falls and the type of fall-related injuries. The location of the fall was categorized as indoors (inside a residence or non-residential building) or outdoors (outside a residence or building, including the driveway/yard and the street). The patient’s activity prior to the fall was categorized as described previously [12].

### Outcomes

The composite outcome of functional decline and/or mortality, 3 and 12 months after the ED visit, was the primary outcome. Functional status at 3- and 12 months were compared to baseline functional status, 2 weeks before the ED visit. Functional decline was defined as at least one point increase in Katz-ADL score or new institutionalization (higher level of assisted living) [18]. Patients with a maximum Katz-ADL score at baseline, institutionalization at baseline, or patients who were lost to follow up were considered as having no functional decline. This assumption was made on the basis of previously executed sensitivity analyses [24].

### Statistical analyses

Data are presented as means with standard deviation (SD), medians with interquartile ranges (IQRs) or numbers with percentages. Differences in patient characteristics between groups were assessed using the Mann-Whitney U test for continuous skewed data and the χ^2^ test for categorical data.

Cohen’s Kappa (κ) was used to quantify the inter-rater reliability of the categorization. Agreement was considered moderate (κ = 0.60–0.79), strong (κ = 0.80–0.90) or almost perfect (κ > 0.90) [25].

Multivariable logistic regression analysis was used to assess the association between patient- and fall characteristics and 3- and 12-months adverse outcomes. First, it was assessed whether an interaction existed between a high risk result according to APOP screening and the cause or location of the fall. This was done by adding an interaction term in the model, and by performance of two separate multivariable regression analyses in which patients were stratified by their geriatric screening result. If there was no interaction between a high risk geriatric screening result and cause or location of the fall, they were possible independent predictors of adverse outcomes, and could both be included in the model. Patient characteristics (age, sex and high risk geriatric screening result) and fall characteristics (cause - and location of fall) were forced into the regression model. Models taking either the cause or the location of the fall into account were executed because of possible multicollinearity. Patients with an unknown cause of the fall (categorized as missing data) were excluded from the multivariable regression analyses on the cause of the fall. Fall-related injuries were not put in the models together with fall characteristics because injuries were in the causal pathway of events and did therefore not meet the criteria of a confounder. In addition, because fall-related injuries (e.g. hip fracture) have already been shown to be associated with adverse outcomes, and fall characteristics were the variables of interest in this study, we did not put fall-related injuries in the multivariable regression models.

Results are presented as odds ratios (ORs) or adjusted odds ratios (AORs) with 95% confidence intervals (CIs). A *p*-value < 0.05 was considered as statistically significant. Statistical analyses were performed using IBM SPSS Statistics version 25.

## Results

**Fig. 1:**
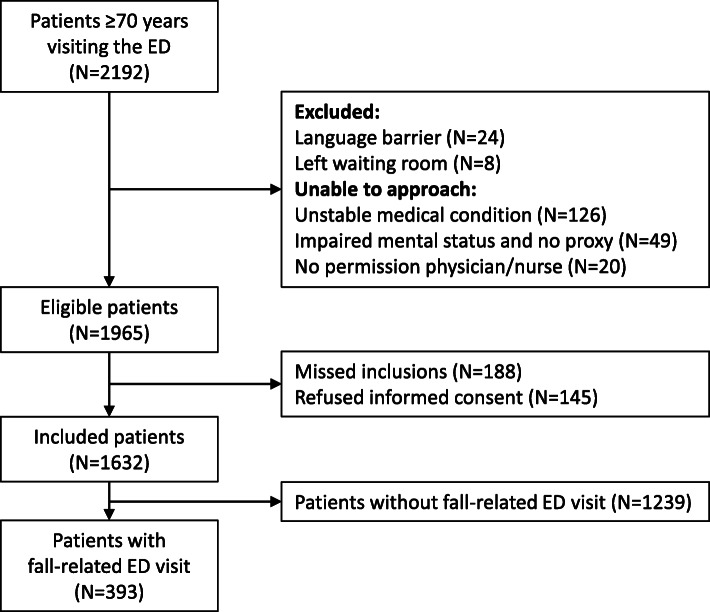
Flowchart of study population. Legend: In total 2192 patients aged ≥70 years visited the EDs of the two hospitals during the inclusion periods. A total of 1965 (89.6%) patients were found eligible of whom 1632 (83.1%) patients were included in the study. Of 1632 included patients 393 (24.1%) patients had a fall-related ED visit and 1239 (75.9%) patients visited the ED without a fall-related problem. Whether the ED visit was fall related was obtained by asking the patient the question: “Is the reason for your ED visit related to a fall?”. After careful retrospective review of the medical files 10 patients switched to the group of patients without a fall-related ED visit

Of the 2192 ED patients aged ≥70 years, 1965 (89.6%) patients were found eligible, of whom 1632 (83.1%) patients were included. A subset of 393 (24.1%) patients with a fall-related ED visit were included in the present study (Fig. 1). The categorization of causes of falls resulted in 87.0% agreement and a strong inter-rater reliability (κ = 0.802) (Additional file 2).

Patient characteristics, circumstances of falls and fall-related injuries for the total study population and stratified by cause of the fall are presented in Table 1. The median age of the overall population was 80 years (IQR 76–86) and 150 (38.2%) patients were male. In total, 238 (60.6%) patients arrived by ambulance and 299 (76.1%) patients had minor trauma as their chief complaint. In total, 193 (49.1%) patients used a walking device and 90 (23.0%) patients were high risk according to the APOP screener.

**Table 1:** Patient characteristics, the circumstance of the fall and fall-related injuries for the total population of patients with a fall-related ED visit and stratified by cause of the fall

	All (***N*** = 393)	Cause of fall		
		Extrinsic cause (***N*** = 195)	Intrinsic cause (***N*** = 115)	Unexplained fall (***N*** = 25)
**Patient characteristics**				
Age (years), median (IQR)	80 (76–86)	80 (75–86)	80 (74–86)	81 (77–88)
Male, n (%)	150 (38.2)	66 (33.8)	51 (44.3)	10 (40.0)
Living independently, n (%)	345 (87.8)	180 (92.3)	92 (80.0)	21 (84.0)
Arrival by ambulance, n (%)	238 (60.6)	114 (58.5)	82 (71.3)	15 (60.0)
Triage urgency, n (%)				
> 1 h (green)	140 (35.6)	81 (41.5)	26 (22.6)	6 (24.0)
< 1 h (yellow)	214 (54.5)	102 (52.3)	71 (61.7)	16 (64.0)
< 10 min (orange)	39 (9.9)	12 (6.2)	18 (15.7)	3 (12.0)
Chief complaint, n (%)				
Minor trauma	299 (76.1)	176 (90.3)	62 (53.9)	12 (48.0)
Malaise	23 (5.9)	3 (1.5)	16 (13.9)	3 (12.0)
Loss of consciousness	33 (8.4)	2 (1.0)	24 (20.9)	7 (28.0)
Others	38 (9.7)	14 (7.2)	13 (11.3)	3 (12.0)
Treating specialism, n (%)				
Surgery	251 (63.9)	158 (81.0)	48 (41.7)	8 (32.0)
Internal medicine	54 (13.7)	10 (5.1)	27 (23.5)	5 (20.0)
Others	88 (22.4)	27 (13.8)	40 (34.8)	12 (48.0)
Use of walking device, n (%)	193 (49.1)	74 (38.3)	68 (59.1)	14 (56.0)
Polypharmacy, n (%)^a^	192 (48.9)	90 (46.2)	57 (49.6)	14 (56.0)
Katz ADL score, median (IQR)	0 (0–1)	0 (0–1)	0 (0–2)	1 (0–2)
6-CIT score, median (IQR)	6 (2–11)	4 (2–8)	8 (4–17)	7 (3–13)
APOP screening result, n (%)				
Low risk	301 (77.0)	164 (85.0)	77 (67.0)	19 (76.0)
High risk	90 (23.0)	29 (15.0)	38 (33.0)	6 (24.0)
**Circumstance of fall**				
Location of fall, n (%)				
Indoors	195 (61.3)	69 (41.8)	83 (83.0)	18 (81.8)
Outdoors	123 (38.7)	96 (58.2)	17 (17.0)	4 (18.2)
Activity prior to fall, n (%)				
Walking	110 (39.6)	81 (43.8)	25 (42.4)	3 (25.0)
Cycling/driving (mobility) scooter	46 (16.5)	45 (24.3)	1 (1.7)	0 (0.0)
Walking up/down stairs/stairlift	28 (10.1)	15 (8.1)	6 (10.2)	3 (25.0)
Getting in/out bed/chair/couch/bath	25 (9.0)	9 (4.9)	10 (16.9)	1 (8.3)
Going to the toilet	15 (5.4)	1 (0.5)	8 (13.6)	3 (25.0)
Exercise	10 (3.6)	8 (4.3)	1 (1.7)	0 (0.0)
Others	44 (15.8)	26 (14.1)	8 (13.6)	2 (16.7)
**Fall-related injuries**^b^				
Type of injury, n (%)				
Minor injury	163 (41.5)	106 (54.4)	29 (25.2)	7 (28.0)
Head injury	135 (34.4)	75 (38.5)	34 (29.6)	12 (48.0)
Fracture	186 (47.3)	121 (62.1)	35 (30.4)	5 (20.0)
Hip fracture	53 (13.5)	32 (16.4)	16 (13.9)	1 (4.0)
No injury	57 (14.5)	3 (1.5)	39 (33.9)	9 (36.0)
Location of injury, n (%)				
Head/face	60 (17.9)	18 (9.4)	24 (31.6)	8 (50.0)
Thorax/abdomen/spine	14 (4.2)	5 (2.6)	2 (2.6)	1 (6.3)
Upper extremity	72 (21.4)	46 (24.0)	13 (17.1)	0 (0.0)
Lower extremity	102 (30.4)	57 (29.7)	24 (31.6)	3 (18.8)
Multiple locations	88 (26.2)	66 (34.4)	13 (17.1)	4 (25.0)

Abbreviations: *N* Number, *IQR* Interquartile range, *ADL* Activities of daily living, *6-CIT* Six-item cognitive impairment test, *APOP* Acutely Presenting Older Patient screening

^a^≥5 self-reported medications

^b^Numbers do not add up to 100% because some people had multiple types and locations of injuries

Missings: 58 cause of the fall, 2 use of walking device, 4 Katz ADL score, 40 6-CIT score, 2 APOP screening result, 75 location of fall, 115 activity prior to fall

In 195 (49.6%) patients the cause of the fall was extrinsic, in 115 (29.3%) patients intrinsic, in 25 (6.4%) patients unexplained and in 58 (14.8%) patients data was missing. Patients with an extrinsic cause most often had minor trauma (90.3%) and were treated by surgeons (81.0%), while patients with an intrinsic- or unexplained cause also presented with malaise and loss of consciousness and were treated by other specialists. Differences in geriatric parameters were observed between the distinct fall groups. A walking device was used in 38.3% of patients with extrinsic causes, compared to 59.1% in intrinsic causes and 56.0% in unexplained falls. In total 15.0% of patients with extrinsic causes had a high risk screening result, compared to 33.0% with an intrinsic cause and 24.0% with an unexplained fall. The location of the fall was indoors for 195 (61.3%) patients. Patients with an extrinsic cause most often fell outdoors (58.2%), while patients with an intrinsic cause most often fell indoors (83.0%). Almost all patients who fell during cycling, driving scooter or exercise had an extrinsic cause of their fall. Patients with intrinsic causes often were walking up/down the stairs (10.2%), getting in/out of bed (16.9%) or were going to the toilet (13.6%). In total, 57 (14.5%) patients had no fall injury, 186 (47.3%) patients had a fracture and 53 (13.5%) patients had a hip fracture. Of the patients with an extrinsic cause 1.5% had no injury, compared to 33.9% in intrinsic causes and 36.0% in unexplained falls.

Patient characteristics stratified by location of the fall are presented in Additional file 3. More patients who fell indoors were considered to have a high risk geriatric screening result compared to patients who fell outdoors (34.9% vs. 4.9%, *p* < 0.001).

Of all 393 patients with fall-related ED visits 26 (6.6%) patients had died and 107 (27.2%) patients experienced functional decline at 3 months follow-up. After 12 months, 61 (15.5%) patients had died and 90 (22.9%) patients experienced functional decline.

The interaction terms for ‘high risk geriatric screening result’, ‘cause of the fall’ and ‘location of the fall’ were all non-significant. Multivariable regression analyses for 3- and 12-months adverse outcomes stratified by geriatric screening result show that there was no effect modification by geriatric screening result and cause or location of the fall (Additional file 4). These results showed that the geriatric screening result was a potential independent predictor of the outcome. A high risk geriatric screening result was associated with an increased risk of adverse outcomes at 3 (AOR 2.27 (1.29–3.98)) and 12 months (AOR 2.20 (1.25–3.89)), adjusted for age and sex. In Table 2, it is shown that adverse outcomes depend on fall characteristics and geriatric screening result. Compared to an extrinsic cause, an intrinsic cause increased the odds for 3-months adverse outcomes independent of a high risk geriatric screening result (AOR 1.92 (1.13–3.26). The cause of the fall was no predictor of 12-months adverse outcomes. A fall indoors, compared to outdoors, was a risk factor for adverse outcomes at 3- (AOR 2.14 (1.22–3.74)) and 12-months (AOR 1.78 (1.03–3.10)) independent of a high risk geriatric screening result.

**Table 2:** Risk on adverse outcomes at 3 and 12 months in older patients with fall-related ED visits depending on fall characteristics and geriatric screening result

	Risk^a^		Risk independent of high risk geriatric screening result^b^	
	3 months	12 months	3 months	12 months
**Cause of fall**				
Extrinsic fall (*n* = 195)	ref	ref	ref	ref
Intrinsic fall (*n* = 115)	2.28 (1.37–3.81)	1.46 (0.88–2.42)	1.92 (1.13–3.26)	1.21 (0.71–2.06)
Unexplained fall (*n* = 25)	2.41 (1.00–5.82)	1.34 (0.55–3.28)	2.29 (0.94–5.57)	1.28 (0.52–3.18)
**Location of fall**				
Outdoors (*n* = 123)	ref	ref	ref	ref
Indoors (*n* = 195)	2.39 (1.39–4.11)	2.01 (1.19–3.41)	2.14 (1.22–3.74)	1.78 (1.03–3.10)

Multivariable logistic regression analyses for the composite outcome of functional decline and/or mortality. Numbers represent Odds Ratios with 95% Confidence Intervals

^a^Model adjusted for age and sex

^b^Model adjusted for age, sex and high risk geriatric screening result

Interaction terms ‘high risk geriatric screening result’*'cause of the fall' and ‘high risk geriatric screening result’*'location of the fall' were not significant

## Discussion

Older patients with a fall-related ED visit represent a heterogeneous group in patient- and fall characteristics. A minority of patients have a high risk on adverse outcomes according to geriatric screening. Apart from the geriatric screening result, both the cause and location of the fall are independent risk factors of 3- and 12-months adverse outcomes.

We described characteristics and outcomes of different types of falls among older patients presenting to the ED. In an overview of 12 large studies evaluating causes of falls in older people, accidents or falls stemming from environmental hazards comprised the largest fall cause category, accounting for 25 to 45%, [10] comparable to the 50% in our category ‘extrinsic cause’. One study found that 9% of falls in older ED patients were caused by syncope, comparable to the 11% found in our study [5]. In the present study we showed that patients who fell indoors were older and had more geriatric impairments in both ADL and cognition compared to patients who fell outdoors, correspond to previous studies [26, 27]. Our findings on adverse outcomes in the total group of older ED patients with falls are comparable with literature [3, 4, 28]. This is the first study that compared functional decline and mortality 3- and 12-months after the ED visit between different types of falls.

A new finding of our study is the large difference in adverse outcomes among patients with different fall characteristics. Categorizing falls into different causes can be arbitrary due to the multifactorial causality and one might even argue that there is no such a thing as an extrinsic or mechanical fall [29]. Therefore, we also categorized patients into different fall circumstances (eg. the location). A minority of older patients with fall-related ED visits were at high risk according to screening, suggesting that it is not only frailty that causes falls [30]. Although we expected otherwise, we found that there was no interaction between the geriatric screening result and cause or location of the fall, indicating that the screening result did not increase the impact of cause or location of the fall. Apart from the geriatric screening result, cause and location of the fall are independent risk factors of adverse outcomes. This could be explained by the observation that older patients who fell indoors and were not screened as ‘frail’ were in some sort of ‘pre-frail’ phase that was not picked up with geriatric screening. It is also possible that the use of other screening tools, known to have different predicting values, may have resulted in slightly different classifications of ‘frail’ vs. ‘non-frail’ patients, i.e. in patients with indoor falls, but it is unlikely that this would have resulted in large differences in the association between location of fall and adverse outcomes. The cause of the fall was an independent predictor for 3-months adverse outcomes, but not for 12-months outcomes, suggesting that location of the fall and the geriatric screening result are more important for predicting long term outcomes. Because fall-related injuries were in the causal pathway of events, we did not correct for injuries as a confounder in the models, but when we did, the results remained the same.

The present study has clinical implications for clinicians in the ED. Current fall risk assessments are complex and time-consuming, [31] but our results suggests that a simple geriatric screening, and assessing the location of the fall already provides important prognostic information. Patient who are high risk according to geriatric screening, fall indoors or have an intrinsic- or unexplained cause may benefit from further fall assessments and interventions. Several geriatric risk stratification tools for the ED setting exists, and although none of them has great predictive power, [8] they might enhance our awareness and understanding of geriatric patients beyond their presenting complaint. Additionally, our data suggests that it remains important to unravel the cause of a fall to start interventions that possibly prevent future falls and adverse outcomes. Adding additional information from the hospital and the home situation, e.g. level of physical activity in everyday life, may further improve clinical prediction tools and tailored decision making [32]. Patients who are at high risk according to geriatric screening and their fall characteristics, could benefit from further assessments on geriatric domains and the risk of future falls by using a comprehensive geriatric assessment (CGA), which has known positive effects on patient outcomes [33]. If is not feasible to execute CGA in the ED, hospitalized patients could be assessed during admission on the ward, and discharged patients could be assessed later by a general practitioner or geriatrician in an outpatient clinic.

This study has several strengths, like a broad unselected population of older ED patients with falls and the multicenter design. There are also several limitations. First, we used self-reported reasons for ED visits to select older patients with falls, which possibly resulted in some missed inclusions. Second, there is no universal categorization of causes of falls, which limits the comparability of our findings. Additionally, the retrospective categorization of causes of falls was complicated by incompleteness of descriptions in medical records. However, terminology from literature was used to design the categories and the inter-rater reliability between researchers was good. Third, the APOP screening instrument was used to measure a proxy of ‘frailty’, while this is technically not a frailty screener but a risk stratification instrument.

## Conclusion

A high risk geriatric screening result and fall characteristics were both independently associated with adverse outcomes in older patients with a fall-related ED visit, suggesting that information on both should be evaluated to guide follow-up geriatric assessment and interventions in clinical care.

## Supplementary Information

**Additional file 1.** Categorization of causes of falls.

**Additional file 2.** Results of categorization of causes of falls.

**Additional file 3.** Patient characteristics and cause of the fall stratified by location of the fall.

**Additional file 4.** Multivariable regression analysis with adverse outcomes at 3 and 12 months in older patients with fall-related ED visits stratified by the result from APOP screening.

## Data Availability

The datasets used and analyzed during the current study are available from the corresponding author on reasonable request.

## Abbreviations

6CIT: 6-item Cognitive Impairment Test
ADL: Activities of Daily Living
AOR: Adjusted Odds Ratio
APOP: Acutely Presenting Older Patient
CI: Confidence Intervals
ED: Emergency Department
IQR: Interquartile Ranges
MTS: Manchester Triage System
OR: Odds Ratio
SD: Standard Deviation
