# Supplementary material for: Geriatric screening, fall characteristics and 3- and 12 months adverse outcomes in older patients visiting the emergency department with a fall
Source: Scand J Trauma Resusc Emerg Med. 2021 Mar 4;29:43. doi: 10.1186/s13049-021-00859-5 (PMC7934471; doi:10.1186/s13049-021-00859-5)
Supplement: Supplementary file 1 — Additional file 1. Categorization of causes of falls. [file 13049_2021_859_MOESM1_ESM.docx]

| **Additional file 1.** Categorization of causes of falls | |
| --- | --- |
| **1. Extrinsic cause** | The patient record describes a cause of the fall that is not medical or intrinsic. This can include i.e. slipping, traffic accidents or alcohol use. |
| 1. *Slip/trip* | A fall due to slipping and/or tripping. This excludes falls with a walking device. |
| 1. *Traffic accident* | A fall as a result of a traffic accident or ascending a certain vehicle. Accidents while cycling are included in this group. |
| 1. *Walking device* | A fall with or because of a walking device. This excludes wheelchairs. |
| 1. *Fall out of bed or (wheel)chair* | A fall out of a bed or (wheel)chair. |
| 1. *Exercise* | A fall during exercise like tennis or football. Falls during cycling are excluded and included in *“extrinsic* – *traffic accidents”.* |
| 1. *Balance* | A fall because of losing balance. This loss of balance is transient and not chronically present, otherwise patients are included in *“intrinsic – gait/balance”.* |
| 1. *Other* | Other causes of an extrinsic fall, including i.e. alcohol consumption. |
| **2. Intrinsic cause** | The patient record describes a clear medical reason for a fall. This includes i.e. a CVA, gait disorders, neurodegenerative diseases and muscle weakness. Syncope is also included as an intrinsic fall. |
| 1. *Neurodegenerative diseases* | A fall in patients with underlying dementia or other neurodegenerative diseases which are stated in the medical record of the patient. This may include Parkinson’s disease or other forms of cognitive impairment. |
| 1. *CVA/TIA* | A fall due to a CVA or a TIA. Ischemia of the brain is also included. Patients who suffer hemiparesis or gait disorders due to a CVA in the past are excluded from this category and included in *“intrinsic – gait/balance”*. |
| 1. *(near-)syncope* | A fall due to syncope or near-syncope. Possible underlying causes of (near-)syncope include reflex syncope, syncope due to orthostatic hypotension and cardiac syncope (cardiovascular). (Near-) syncope eci is also included. |
| 1. *Gait/balance* | A fall caused by an internal gait- or balance problem. This includes chronic presence of vertigo and dizziness and chronical problems with walking or hemiparesis caused by a CVA in the past. Parkinson’s disease is excluded and included in *“intrinsic – neurodegenerative diseases”.* |
| 1. *Other* | Other intrinsic causes of a fall, including i.e. falls due to chronic muscle weakness or falls in patients with malaise due to internal diseases like pneumonia. |
| **3. Unexplained fall** | No apparent cause of the fall. In the patient record, neither a medical nor a mechanical reason is described. The record must provide a context indicating physician(s) searched for a cause but could not define it or the record must explicitly state that the patient had no recollection of the fall or that history taking was not possible. |
| **4. Missing data** | The record provides no context or cause of the fall. |
