# Supplementary material for: Geriatric screening, fall characteristics and 3- and 12 months adverse outcomes in older patients visiting the emergency department with a fall
Source: Scand J Trauma Resusc Emerg Med. 2021 Mar 4;29:43. doi: 10.1186/s13049-021-00859-5 (PMC7934471; doi:10.1186/s13049-021-00859-5)
Supplement: Supplementary file 2 — Additional file 2. Results of categorization of causes of falls. [file 13049_2021_859_MOESM2_ESM.docx]

**ADDITIONAL FILE 2.**

| **Additional file 2.** Results of categorization of causes of falls | |
| --- | --- |
|  | N (%) |
| **Extrinsic cause** |  |
| *Slip/trip* | 98 (24.9) |
| *Traffic accident* | 50 (12.7) |
| *Walking device* | 9 (2.3) |
| *Fall out of bed or (wheel)chair* | 5 (1.3) |
| *Exercise* | 6 (1.5) |
| *Balance* | 13 (3.3) |
| *Other* | 14 (3.6) |
| **Intrinsic cause** |  |
| *Neurodegenerative diseases* | 27 (6.9) |
| *CVA/TIA* | 11 (2.8) |
| *(near-)syncope* | 44 (11.2) |
| *Gait/balance* | 14 (3.6) |
| *Other* | 19 (4.8) |
| **Unexplained fall** | 25 (6.4) |
| **Missing data** | 58 (14.8) |
