# Supplementary material for: Geriatric screening, fall characteristics and 3- and 12 months adverse outcomes in older patients visiting the emergency department with a fall
Source: Scand J Trauma Resusc Emerg Med. 2021 Mar 4;29:43. doi: 10.1186/s13049-021-00859-5 (PMC7934471; doi:10.1186/s13049-021-00859-5)
Supplement: Supplementary file 3 — Additional file 3. Patient characteristics and cause of the fall stratified by location of the fall. [file 13049_2021_859_MOESM3_ESM.docx]

**ADDITIONAL FILE 3.**

| **Additional file 3.** Patient characteristics and cause of the fall stratified by location of the fall | | | |
| --- | --- | --- | --- |
|  | **Location of fall** | |  |
|  | **Indoors**  **(N=195)** | **Outdoors**  **(N=123)** | **p-value** |
| **Patient characteristics** |  |  |  |
| Age (years), median (IQR) | 82 (76-87) | 78 (74-82) | <0.001 |
| Male, n (%) | 73 (37.4) | 52 (42.3) | 0.389 |
| Living independently, n (%) | 162 (83.1) | 119 (96.7) | <0.001 |
| Arrival by ambulance, n (%) | 138 (70.8) | 74 (60.2) | 0.051 |
| Triage urgency, n (%) |  |  | 0.003 |
| > 1 hour (green) | 47 (24.1) | 52 (42.3) |  |
| < 1 hour (yellow) | 128 (65.6) | 61 (49.6) |  |
| < 10 min (orange) | 20 (10.3) | 10 (8.1) |  |
| Chief complaint, n (%) |  |  | 0.230 |
| Minor trauma | 138 (70.8) | 96 (78.0) |  |
| Malaise | 15 (7.7) | 4 (3.3) |  |
| Loss of consciousness | 23 (11.8) | 10 (8.1) |  |
| Others | 19 (9.7) | 13 (10.6) |  |
| Treating specialism, n (%) |  |  | 0.054 |
| Surgery | 109 (55.9) | 93 (75.6) |  |
| Internal medicine | 28 (14.4) | 11 (8.9) |  |
| Others | 58 (29.7) | 19 (15.4) |  |
| Use of walking device, n (%) | 126 (64.6) | 28 (23.0) | <0.001 |
| Polypharmacy, n (%) | 106 (54.4) | 54 (43.9) | 0.069 |
| Katz ADL score, median (IQR) | 1 (0-2) | 0 (0-0) | <0.001 |
| 6-CIT score, median (IQR) | 8 (4-15) | 4 (0-8) | <0.001 |
| APOP screening result, n (%) |  |  | <0.001 |
| Low risk | 127 (65.1) | 116 (95.1) |  |
| High risk | 68 (34.9) | 6 (4.9) |  |
|  |  |  |  |
| **Cause of fall** |  |  | <0.001 |
| Extrinsic cause | 69 (35.4) | 96 (78.0) |  |
| Intrinsic cause | 83 (42.6) | 17 (13.8) |  |
| Unexplained fall | 18 (9.2) | 4 (3.3) |  |
| Abbreviations: N=number; IQR=interquartile range; ADL=activities of daily living; 6-CIT=six-item cognitive impairment test; APOP=Acutely Presenting Older Patient screening.  Missings: 75 location of fall, 1 walking device, 2 Katz ADL, 31 6-CIT; 1 APOP screening result, 31 cause of fall | | | |
