# Supplementary material for: Geriatric screening, fall characteristics and 3- and 12 months adverse outcomes in older patients visiting the emergency department with a fall
Source: Scand J Trauma Resusc Emerg Med. 2021 Mar 4;29:43. doi: 10.1186/s13049-021-00859-5 (PMC7934471; doi:10.1186/s13049-021-00859-5)
Supplement: Supplementary file 4 — Additional file 4. Multivariable regression analysis with adverse outcomes at 3 and 12 months in older patients with fall-related ED visits stratified by the result from APOP screening. [file 13049_2021_859_MOESM4_ESM.docx]

**ADDITIONAL FILE 4.**

| **Additional file 4.** Multivariable regression analysis with adverse outcomes at 3 and 12 months in older patients with fall-related ED visits stratified by the result from APOP screening | | | | | | |
| --- | --- | --- | --- | --- | --- | --- |
|  |  | **Risk of adverse outcome**  **at 3 months** | |  | **Risk of adverse outcome**  **at 12 months** | |
|  |  | **Low risk geriatric screening result** | **High risk geriatric screening result** |  | **Low risk geriatric screening result** | **High risk geriatric screening result** |
|  |  | **OR (95% CI)** | **OR (95% CI)** |  | **OR (95% CI)** | **OR (95% CI)** |
| **Patient characteristics** |  |  |  |  |  |  |
| Age |  | 1.05 (1.00-1.11) | 1.05 (0.96-1.16) |  | 1.10 (1.04-1.17) | 1.08 (0.97-1.21) |
| Male |  | 0.86 (0.46-1.62) | 0.62 (0.21-1.87) |  | 1.47 (0.79-2.75) | 0.90 (0.27-3.07) |
|  |  |  |  |  |  |  |
| **Fall characteristics** |  |  |  |  |  |  |
| *Cause of fall* |  |  |  |  |  |  |
| Extrinsic fall |  | ref | ref |  | ref | ref |
| Intrinsic fall |  | 1.87 (0.92-3.81) | 0.81 (0.27-2.42) |  | 1.49 (0.73-3.04) | 0.41 (0.12-1.43) |
| Unexplained fall |  | 1.83 (0.61-5.55) | 1.16 (0.15-9.16) |  | 0.76 (0.23-2.54) | 1.20 (0.10-15.04) |
| *Location of fall* |  |  |  |  |  |  |
| Outdoors |  | ref | ref |  | ref | ref |
| Indoors |  | 2.10 (1.06-4.13) | 0.79 (0.11-5.89) |  | 2.21 (1.12-4.34) | 0.57 (0.05-6.48) |
| Abbreviations: OR= Odds ratio; CI= confidence interval. | | | | | | |
